# Effect of Porcine-Derived Collagen Membrane Crosslinking on Intraoral Soft Tissue Augmentation: A Canine Model

**DOI:** 10.3390/bioengineering12080875

**Published:** 2025-08-14

**Authors:** Blaire V. Slavin, Vasudev Vivekanand Nayak, Zachary M. Stauber, Quinn T. Ehlen, Joseph P. Costello, Orel Tabibi, Justin E. Herbert, Ricky Almada, Sylvia Daunert, Lukasz Witek, Paulo G. Coelho

**Affiliations:** 1 University of Miami Miller School of Medicine, Miami, FL 33136, USA; 2 Department of Biochemistry and Molecular Biology, University of Miami Miller School of Medicine, Miami, FL 33136, USA; 3 Dr. John T. Macdonald Foundation Biomedical Nanotechnology Institute (BioNIUM), University of Miami, Miami, FL 33136, USA; 4 Biomaterials and Regenerative Biology Division, NYU College of Dentistry, New York, NY 10010, USA; 5 Department of Biomedical Engineering, NYU Tandon School of Engineering, Brooklyn, NY 11201, USA; 6 Hansjörg Wyss Department of Plastic Surgery, NYU Grossman School of Medicine, New York, NY 10016, USA; 7 Department of Oral and Maxillofacial Surgery, NYU College of Dentistry, New York, NY 10010, USA; 8 DeWitt Daughtry Family Department of Surgery, Division of Plastic & Reconstructive Surgery, University of Miami Miller School of Medicine, Miami, FL 33136, USA; 9 Sylvester Comprehensive Cancer Center, University of Miami Miller School of Medicine, Miami, FL 33136, USA

**Keywords:** collagen membrane, crosslinking, dental implant, keratinized tissue, soft tissue augmentation

## Abstract

Peri-implant disease and gingival recession may be partially attributed to inadequate keratinized tissue. Soft tissue augmentation procedures utilizing non-autologous biomaterials, such as porcine-derived collagen membranes, have been gaining prominence and exogenous crosslinking is being actively investigated to improve the collagen membrane’s stability and potential for keratinized tissue gain. The aim of this preclinical study was to evaluate the performance of a novel, crosslinked porcine collagen membrane (Zderm^TM^, Osteogenics Biomedical, Lubbock, TX, USA) relative to an established, commercially available, non-crosslinked counterpart (Mucograft^®^, Geistlich Pharma North America Inc., Princeton, NJ, USA) in a canine mandibular model. Bilateral split-thickness mucosal defects were created in adult beagles (*n* = 17), with each site receiving one membrane. Qualitative and quantitative histomorphometric analyses of groups were performed after 4, 8, and 12 weeks of healing and compared to unoperated, positive controls from the same subject. No significant differences in membrane presence were observed between Zderm^TM^ and Mucograft^®^ at 4, 8, and 12 weeks of permitted healing (*p* > 0.05). Similarly, the average keratinized tissue (KT) length between Zderm^TM^ and Mucograft^®^ groups was statistically equivalent across all healing times (*p* > 0.05). However, qualitative histological evaluation revealed greater rete ridge morphology amongst defects treated with Zderm^TM^ in comparison to Mucograft^®^. Nevertheless, both membranes exhibited excellent biocompatibility and are well-suited for soft tissue augmentation procedures in the oral cavity.

## 1. Introduction

The presence of adequate keratinized mucosa is vital in the maintenance of periodontal and peri-implant health. Reduced peri-implant keratinized mucosa has been described as a potential contributor to peri-implant tissue breakdown with subsequent concern for the development of peri-implant mucositis, peri-implantitis, and eventual implant failure [1,2,3]. Similarly, accelerated gingival recession is believed to be a consequence of inadequate keratinized gingiva with concern for dentinal sensitivity, root caries, alveolar bone, and tooth loss [3,4]. Innovative strategies for soft tissue augmentation have advanced over recent decades to combat the disruption of this crucial biologic seal. While the autologous free gingival graft and subepithelial connective tissue graft are potential solutions, their associated donor site morbidity, increased operative time, patient-reported pain, and esthetic dissatisfaction with respect to color and texture remain problematic [5,6]. Non-autologous alternatives, such as the xenogeneic collagen membrane, continue to be investigated as potential comparable alternatives without the aforementioned drawbacks [3,7,8].

As the most abundant extracellular matrix protein in the human body, collagen’s inherent biological, mechanical, and structural properties facilitate cellular attachment and differentiation [9]. Preclinical studies that have examined the behavior of a porcine-derived collagen membrane intraorally revealed that the membranes elicited a short inflammatory phase followed by the invasion of vimentin-positive cells, implicated in both collagen production and revascularization [10,11]. For example, Mucograft^®^ (Geistlich Pharma North America Inc., Princeton, NJ, USA) is one such collagen membrane composed of a non-permeable outer layer and porous inner layer, that has well-established efficacy in achieving tissue keratinization [12,13,14,15,16,17]. A recent randomized controlled clinical trial that compared the use of Mucograft^®^ to connective tissue grafts found adequate and sustained keratinized tissue gain at 6 months postoperatively, significantly improved esthetic appearance, less operative time, and lowered pain in the xenograft group [18]. However, a direct comparison of keratinized tissue formation and rete ridge development in the oral mucosa between the non-crosslinked Mucograft^®^ and a crosslinked porcine-derived membrane alternative remains largely unexplored.

The objective of this study was to evaluate the in vivo performance of crosslinked and non-crosslinked bilayered porcine-derived collagen membranes in a large translational (canine) mandibular model. By assessing biocompatibility, biodegradation patterns, and gain of keratinized tissue length, this research aimed to elucidate whether exogenous crosslinking influences healing outcomes with the use of collagen membranes in soft tissue augmentation procedures. The postulated null hypothesis was that membrane crosslinking would not affect rete ridging architecture, membrane presence, and keratinized tissue formation at any of the observed study timepoints.

## 2. Materials and Methods

### 2.1. Surgical Protocol Summary

The study was approved by the institutional animal care and use committee of North American Science Associates LLC (approval number: IAN004-IS75). According to a sample size calculation for statistical power greater than 0.8, an effect size of 0.7, and type I error frequency (α) of 0.05, *n* = 33 defects were required to address the study hypothesis. Subjects included adult beagle dogs (~1.5 years) in good health. Subjects were allowed to acclimate to the surgical facility, NAMSA, for 7 days prior to any surgical intervention. Animals were fasted for at least 12 hours on the day of surgery in preparation for general anesthesia. Animals were anesthetized with an intramuscular injection of midazolam (0.1–0.2 mg/kg) and butorphanol (0.05–0.1 mg/kg), and anesthesia was maintained using isoflurane (2–5%) via intubation tube and intravenous propofol (2–8 mg/kg). Prior to surgical intervention, an additional bilateral caudal mandibular block using bupivacaine (0.5–2.0 mg/kg) was administered.

A split mucosal flap was raised, and the submucosal connective tissue partially removed, to create bilateral soft tissue mandibular defects with the following dimensions: 10 × 5 mm - length (anterior–posterior) × height (superior-inferior). Each defect was treated either with (i) crosslinked porcine-derived collagen membrane (Zderm^TM^: Osteogenics Biomedical, Lubbock, TX, USA) or (ii) non-crosslinked porcine-derived collagen membrane (Mucograft^®^: Geistlich Pharma North America Inc., Princeton, NJ, USA). Membranes were left exposed and sutured using polytetrafluoroethylene sutures (Cytoplast™ Non-Absorbable PTFE Sutures, Osteogenics Biomedical, Lubbock, TX, USA).

Intramuscular injections of buprenorphine (0.05 mg/kg), tramadol (4–10 mg/kg), carprofen (2.5–5.0 mg/kg) were administered for pain in addition to cefpodoxime (5–10 mg/kg) in the immediate postoperative period. Incision sites were monitored by trained staff for healing, and animals were provided food and water regularly. Subjects (*n* = 5 at 4 and 8 weeks, and *n* = 7 at 12 weeks) were euthanized at their respective timepoints, followed by the harvesting of their mandibles, which were immediately immersed in 10% formalin. These end points were based on a previous study where palatal injury in beagles treated with skin-derived dermal substrates showed transition of thin collagen fibers with inflammatory cell presence at the early healing timepoints to well-aligned collagen fiber networks and presence of rete ridges after 12 weeks [19].

### 2.2. Histological Processing

The samples were gradually dehydrated in a series of ethanol solutions ranging from 70 to 100%, and embedded in a methacrylate-based resin. Samples were oriented along the buccal–lingual axis and sliced (~250 μm thick) in a mesial–distal direction with a precision diamond wafering saw (Isomet 2000, Buehler Ltd., Lake Bluff, IL, USA). Individual slices were then glued to acrylic plates using a cyanoacrylate-based adhesive (Loctite 408, Henkel AG, Dusseldorf, Germany) and left to set for 24 h. A final thickness of ~80 μm was achieved by running samples over a series of silicon carbide (SiC) abrasive papers (400, 600, 800, and 1200; Buehler Ltd., Lake Bluff, IL, USA) under copious irrigation on a grinding/polishing machine (Metaserv 3000, Buehler Ltd., Lake Bluff, IL, USA).

Samples were stained with Stevenel’s Blue and Van Gieson’s picrofuchsin (SVG). Stevenel’s blue-stained cells and extracellular structures in a gradation of blue tones. Van Gieson’s picrofuchsin, the counterstain, stained collagen fibers green, bone in red, and muscle fibers in blue. This staining combination allowed for the differentiation of soft, connective, osteoid, and mineralized tissues. An automated slide scanning system and a specialized computer software (Aperio CS2 and ImageScope, Leica BioSystems, Deer Park, IL, USA) were utilized to review the histomicrographs.

Qualitative and semi-quantitative analyses were performed by an experienced investigator who was blinded to time in vivo (4, 8, 12 weeks) and treatment group. In both analyses, the morphology of the epithelial layer and lamina propria of defects treated with either membrane was compared to that of unoperated soft tissue sections within the same animal to control for any anatomical variation between subjects.

### 2.3. Qualitative Analysis

Histomicrographs of all defect sites treated with Zderm^TM^ and Mucograft^®^ were reviewed by an experienced investigator and compared to their corresponding positive control soft tissue sections. Since progressive changes in rete ridge depth and architecture are characteristic of the quality of regenerating oral mucosa, qualitative analysis focused on the comparison of rete ridge morphology between groups [20].

### 2.4. Semi-Quantitative Analysis

**Figure 1:**
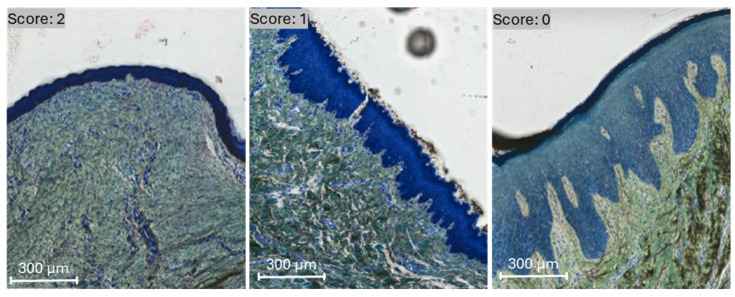
Membrane presence was evaluated and scored by visualization under transmitted light microscopy. A score of 0, 1, and 2 was defined by membrane absence, partial-, and full-presence, respectively, on representative histomicrographs.

Membrane presence was evaluated as an assessment of biodegradation and volume stability. For semiquantitative analysis of membrane presence, each defect site that underwent treatment was ranked using a scale ranging from 0 to 2 (described in Table 1 and illustrated in Figure 1). Scores were compared by experimental group across 4, 8, and 12 week timepoints in vivo. Statistical analyses were performed using the Kruskal–Wallis non-parametric test using SPSS v29 (IBM, Armonk, NY, USA), and the data are presented as medians and interquartile ranges (IQR).

### 2.5. Quantitative Analysis

Histological slides were mounted for quantification of keratinized tissue length (KT length) utilizing ImageJ v 1.54i (U.S. National Institutes of Health, Bethesda, MD, USA). KT length was then analyzed statistically through SPSS v29 (IBM, Armonk, NY, USA). Evaluation of KT length is presented as mean values with the corresponding 95% confidence interval (CI) values (mean  ±  95% CI). KT length values were analyzed using a general linear mixed model approach. All data were assessed for normality using the Shapiro–Wilk test prior to any analysis. The terminal measurements for the histological images of the treated surgical sites were determined.

**Figure 2:**
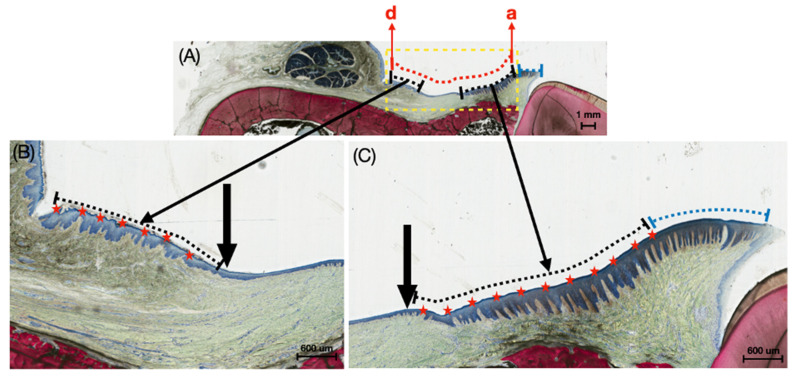
(**A**) Representative histomicrograph of the buccal mucosa with dashed yellow lines indicating the defect boundary, dashed red line representing the total length of the induced defect, dashed black lines highlighting the length of the regenerated keratinized tissue within the induced defect site, and dashed blue lines showing the native keratinized tissue outside the induced defect region. Points ‘a’ and ‘d’ denote the ends of the defect length; (**B**,**C**) higher magnification histomicrographs showing the transition between keratinized tissue (along the dashed black lines) and regenerating buccal mucosa. Black arrows demarcate these transition regions, while red stars indicate the keratinized tissue within the induced defect site, and dashed blue lines show the native keratinized tissue outside the induced defect region.

For the low magnification histological images, sites a–d, depicted in Figure 2, were analyzed. The yellow dashed box demarcates the defect boundary; the red dashed line represents the total length of the defect; the black dashed line shows regenerated KT within the defect site; the blue dashed line shows native KT outside the defect site (Figure 2). A high magnification of site d is also provided for qualitative analysis of keratinized tissue at the KT-host interface.

## 3. Results

### 3.1. Postoperative Observations

No signs of infection, inflammation, or soft tissue disruption were observed. Upon inspection, treatment sites exhibited some discoloration and raised mucosa consistent with tissue healing over biocompatible materials and adjacent suture threads.

### 3.2. Qualitative Histologic Findings

**Figure 3:**
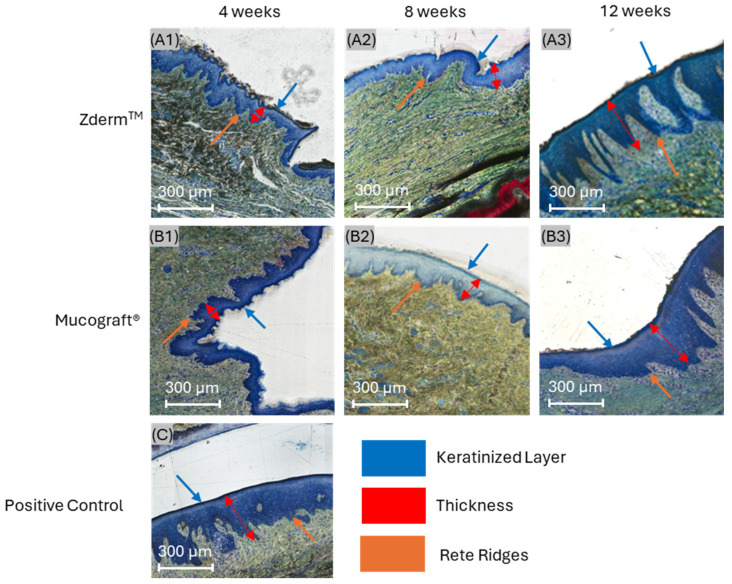
Histomicrographs of soft tissue defects treated with (**A1**–**A3**) Zderm^TM^ (crosslinked) and (**B1**–**B3**) Mucograft^®^ (non-crosslinked) relative to (**C**) unoperated positive controls after 4 (**A1**,**B1**), 8 (**A2**,**B2**), and 12 weeks (**A3**,**B3**) in vivo. Orange, red, and blue arrows highlight rete ridge morphology, epithelial thickness, and keratinized tissue presence, respectively.

Histologic micrographs at 4 weeks demonstrated healing of split-thickness defects with a thin keratinized epithelial layer in groups treated with Zderm^TM^ (Figure 3A1) and Mucograft^®^ (Figure 3B1). At this early time point, rete ridging was absent or minimal relative to unoperated positive controls (Figure 3C), which exhibited a high degree of uniform, undulating rete ridges at the junction between the epidermis and lamina propria. However, the crosslinked group displayed more appreciable rete ridge projection into the connective tissue in comparison to the non-crosslinked group at this time point. Inflammatory cells were distributed throughout the incorporating membrane and in the connective tissue surrounding both membrane groups.

At 8 weeks, a general reduction in inflammatory content was appreciable in both experimental groups. Rete ridges projecting into the underlying lamina propria were more developed in both groups compared to their 4-week counterparts. Notably, the Zderm^TM^ group (crosslinked) exhibited pronounced epithelial thickening and distinctive, uniform rete ridge infiltration at 8 weeks, resembling unoperated positive controls more closely than the Mucograft^®^ (non-crosslinked) group (Figure 3A2,B2).

At 12 weeks, specimens from both treatment groups exhibited a dense, well-organized connective tissue network compared to those at 8 weeks. Capillaries were visible throughout the lamina propria, and inflammatory cell presence was low in both groups, indicating favorable membrane integration and biocompatibility. At this extended time point, rete ridge development was more advanced in defects treated with Zderm^TM^ relative to those treated with Mucograft^®^ (Figure 3A3,B3). Additionally, the epithelial thickness of both treatment groups appeared greater compared to positive controls.

### 3.3. Semi-Quantitative Histologic Findings

**Figure 4:**
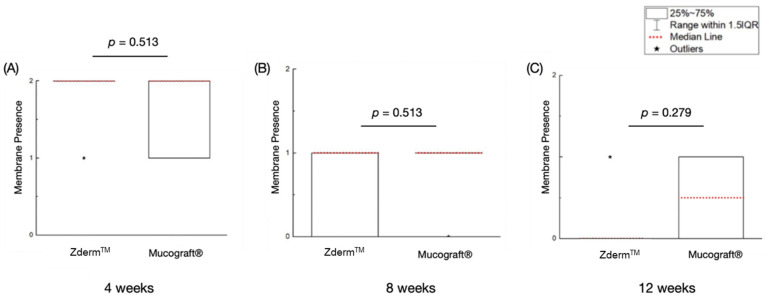
Membrane presence of the treatment groups at (**A**) 4 weeks, (**B**) 8 weeks, and (**C**) 12 weeks of permitted healing. *p* < 0.05 is statistically significant.

Ranked membrane presence values are summarized in Table 2. No significant differences in membrane presence were observed between Zderm^TM^ and Mucograft^®^ at 4 (*p* = 0.513), 8 (*p* = 0.513), and 12 (*p* = 0.279) weeks of permitted healing (Figure 4). Nonetheless, both groups demonstrated a reduction in membrane presence as the healing time progressed, with a return to native tissue morphology by the 12-week timepoint.

### 3.4. Quantitative Histologic Findings

**Figure 5:**
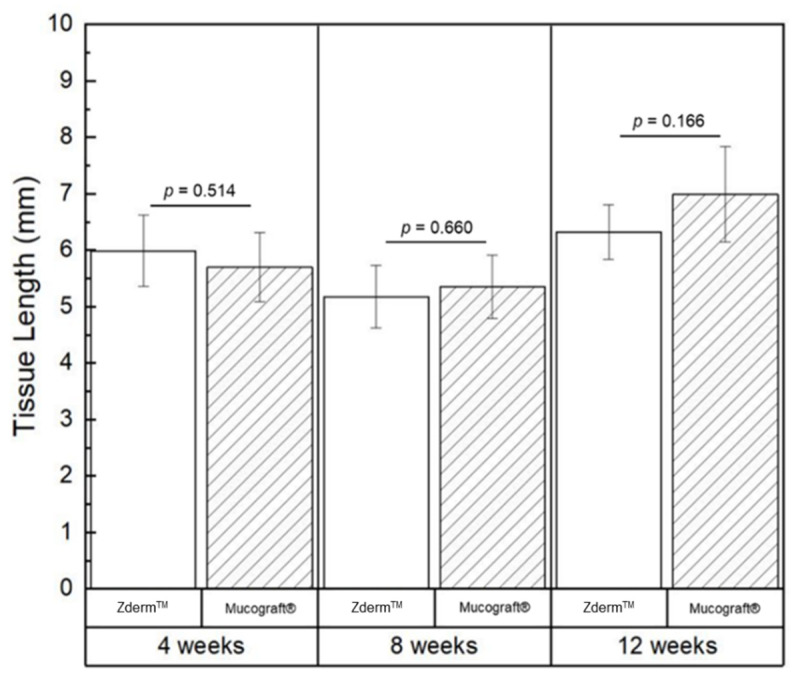
Average measurements of keratinized tissue length depicted graphically, with data shown as means and corresponding 95% confidence intervals. *p* < 0.05 was considered statistically significant.

No significant differences were observed with respect to average keratinized tissue (KT) length between Zderm^TM^ or Mucograft^®^ groups at 4- (*p* = 0.514), 8- (*p* = 0.660), and 12-week (*p* = 0.166) timepoints, as illustrated in Table 3 and Figure 5.

## 4. Discussion

Volume instability and rapid degradation kinetics are two of the primary challenges associated with the implementation of collagen membranes for intraoral soft tissue augmentation [21,22]. The present study sought to compare a novel, crosslinked porcine-derived collagen membrane (Zderm^TM^) to a non-crosslinked membrane (Mucograft^®^). Rete ridge depth and architecture at the junction between regenerating epithelium and lamina propria were greater in the Zderm^TM^ group, particularly after 12 weeks of permitted healing.

The impact of non-crosslinked porcine-derived collagen matrix (Mucograft^®^) on soft tissue regeneration has been examined in previous studies amongst patients with risk factors (smoking and intraoral manifestations of head and neck cancer treated with resection) that poorly impact their healing capacity following periodontal surgery [23,24]. In these cases, Mucograft^®^ improved the width of keratinized tissue, thickness of keratinized gingiva, and permitted complete root coverage after 3 and 6 months [23,24]. Furthermore, the porcine-derived collagen membrane enlarged the width of attached peri-implant gingiva with stability after 6 months [24]. Regarding rete ridge morphology, a separate clinical study demonstrated that the oral epithelium exhibited a keratinized stratum corneum with an established keratin layer after 12 weeks [25]. Although rete ridges were observed with the use of Mucograft^®^, they generally presented with irregular morphology in most instances, similar to the findings of the current study [25]. Rete ridges contribute to the maintenance of epidermal structure and mechanical properties by increasing the contact area between the epidermis and dermis, which enhances shear resistance [26,27]. Engineered epithelial tissue, for example, has been observed to detach from the underlying dermal layers, which may be attributed to the absence of rete ridges compared to native oral mucosa and challenges in replicating interactions between dermal and epidermal components [20]. While there were no appreciable differences in the gain of keratinized tissue length between treatment and control groups, the qualitative improvements in rete ridge morphology associated with the Zderm^TM^ may be due to the differences in membrane crosslinking. This warrants additional investigation into the potential benefits of crosslinking, and the mechanistic investigation of the performance at extended timepoints in vivo [20].

Collagenases possessed by periodontal bacteria, macrophages, and polymorphonuclear leukocytes, compromise membrane stability [21,28]. To maximize their benefits, strategies to improve the mechanical and chemical strength of collagen membranes have been employed. One such strategy is exogenous collagen crosslinking, the formation of covalent bonds between collagen molecules via chemical, physical, or biological processes [9]. Although chemical crosslinking methods yield higher degrees of membrane stabilization, these processes may come at the expense of eliciting an adverse host response that, in turn, compromises native tissue integration [29,30]. While physical and biological methods circumvent the potential for cytotoxicity, they are far weaker than traditional chemical approaches [9]. Cross-linking enhances both tensile strength and resistance to collagenase, thereby extending the membrane’s durability in vivo. This increased stability may provide a consistent cellular signal for the formation of rete ridges [31,32]. Of note, this resistance to degradation has been shown to be dependent on the type of crosslinking utilized [31].

For these reasons, it is imperative that a collagen crosslinker exhibits strong crosslinking capabilities, good cytocompatibility, and membrane degradation kinetics. However, the increase in density associated with high exogenous crosslinking may negatively impact cellular ingrowth, trans membranous vascularization, and elicit a strong foreign body reaction that compromises the membrane’s regenerative potential [33,34]. Thus, while the improved barrier function of a highly crosslinked collagen membrane may be well-suited for guided bone regeneration [35,36,37], it may be undesirable for the purposes of soft tissue augmentation. However, it is important to highlight that the crosslinking process utilized to create Zderm^TM^ did not interfere with the membrane’s cell permeability or its ability to integrate completely into the surrounding native connective tissue after 12 weeks in vivo. While the exact crosslinking methodology of Zderm^TM^ is a trade secret of the manufacturer, future studies should focus on determining the underlying mechanisms by which the observed increase in rete ridge architecture in Zderm^TM^ might affect extracellular remodeling, epithelial–mesenchymal interaction, and fibroblast migration. For instance, rete ridges regulate the population of adult keratinocyte stem cells within the epidermis by sustaining a homeostatic equilibrium between cellular differentiation and renewal [38,39]. Furthermore, the formation of rete ridges is thought to result from regulated interactions between the epithelium and lamina propria [40]. Accordingly, an appropriate biomaterial scaffold, such as a collagen membrane, may facilitate the restoration of rete ridge architecture [40], and it could be possible that the Zderm^TM^ membrane exhibited improved mechanical stability over Mucograft^®^ over 12 weeks, resulting in superior rete ridge architecture.

On a separate note, off-label use of xenogeneic collagen membranes for skin defects has also begun to be explored with favorable results [41,42,43]. Future areas of investigation also include the potential of improved performance of this crosslinked porcine collagen membrane with the addition of biologics or the exploration of its effectiveness in patients at risk for compromised wound healing. For example, the potential to enhance the bioactivity of collagen-based membranes with platelet-rich fibrin has been demonstrated with scientific evidence to enhance soft tissue regeneration [44,45,46]. Altogether, the existing literature prompts future investigation into the potential compatibility and enhancement of Zderm^TM^’s function with these biologics.

## 5. Conclusions

Assessment of keratinized tissue length and membrane degradation pattern of defects treated with the well-established, non-crosslinked (Mucograft^®^) and novel, crosslinked (Zderm^TM^) porcine-derived collagen membranes was statistically homogenous across all timepoints. The qualitative histological analysis validated a return to native morphology of both groups in comparison to unoperated controls. This study had certain limitations, including the 12-week endpoint and a lack of mechanistic evaluation of soft tissue formation, necessitating follow-up evaluation. Within these limitations, no appreciable differences in gain of keratinized tissue length or biocompatibility were observed between membrane groups, with the exception of the qualitative improvements in rete ridge morphology associated with Zderm^TM^.

## Figures and Tables

**Table 1:** Ranking scale used to measure membrane presence in the experimental groups across timepoints.

Membrane Presence	Description
0	No membrane presence characterized by an absence of gaps in the healing layers with a dense, organized connective tissue, and a return to normal structural tissue morphology
1	Partial membrane presence characterized by an absence of gaps in the healing layer and residual membrane (indicating degradation)
2	Full membrane presence characterized by tissue integration with minimal gaps in the healing layer and inflammatory reaction to the membranes

**Table 2:** A descriptive table indicating semi-quantitative membrane presence, inflammation data at 4, 8, and 12 weeks. Data presented as medians and respective interquartile ranges (median (IQR)).

**Membrane Presence**	**Group**	**Median (IQR)**		
		**4 Weeks**	**8 Weeks**	**12 Weeks**
	Zderm^TM^	2 (0)	1(1)	0 (0)
	Mucograft^®^	2 (1)	1 (0)	0.5 (1)

**Table 3:** Average measurements of keratinized tissue length (mm) of the crosslinked (Zderm^TM^) and non-crosslinked (Mucograft^®^) membranes at the 3 different evaluation timepoints. Data presented as means and corresponding 95% confidence intervals.

Timepoint	Zderm^TM^ Average KT Length (mm)	Mucograft^®^ Average KT Length (mm)	*p*-Value
4 weeks	5.99 ± 0.67	5.70 ± 0.66	0.514
8 weeks	5.18 ± 0.60	5.35 ± 0.60	0.660
12 weeks	6.32 ± 0.52	6.99 ± 0.90	0.166

## Data Availability

The raw data supporting the conclusions of this article will be made available by the authors on request.
